# Lumbar puncture simulation in pediatric residency training: improving procedural competence and decreasing anxiety

**DOI:** 10.1186/s12909-016-0722-1

**Published:** 2016-08-08

**Authors:** Hugh J. McMillan, Hilary Writer, Katherine A. Moreau, Kaylee Eady, Erick Sell, Anna-Theresa Lobos, Jenny Grabowski, Asif Doja

**Affiliations:** 1 Department of Pediatrics, Division of Neurology, Children’s Hospital of Eastern Ontario Research Institute, University of Ottawa, 401 Smyth Rd, Ottawa, ON K1H 8L1 Canada; 2 Department of Pediatrics, Division of Critical Care, Children’s Hospital of Eastern Ontario Research Institute, University of Ottawa, 401 Smyth Road, Ottawa, ON K1H 8L1 Canada; 3 Faculty of Education, University of Ottawa, 145 Jean-Jacques-Lussier Private, Ottawa, ON K1N 6N5 Canada; 4 Children’s Hospital of Eastern Ontario Research Institute, 401 Smyth Road, Ottawa, ON K1H 8L1 Canada

**Keywords:** Lumbar puncture, Medical education, Patient simulation, Pediatric, Anxiety, Training program

## Abstract

**Background:**

Pediatric residents must become proficient with performing a lumbar puncture (LP) during training. Residents have traditionally acquired LP skills by observing the procedure performed by a more senior resident or staff physician and then attempting the procedure themselves. This process can result in variable procedural skill acquisition and trainee discomfort. This study assessed changes in resident procedural skill and self-reported anxiety when residents were provided with an opportunity to participate in an interactive training session and practice LPs using a simulator.

**Methods:**

All pediatric residents at our institution were invited to participate. Residents were asked to report their post-graduate year (PGY), prior LP attempts and self-reported anxiety scores as measured by the standardized State-Trait Anxiety Inventory - State Anxiety Scale (STAI-S) prior to completing an observed pre-test using an infant-sized LP simulator. Staff physicians observed and scored each resident’s procedural skill using a previously published 21-point scoring system. Residents then participated in an interactive lecture on LP technique and were given an opportunity for staff-supervised, small group simulator-based practice within 1 month of the pre-test. Repeat post-test was performed within 4 months.

**Results:**

Of the pediatric residents who completed the pre-test (*N* = 20), 16/20 (80 %) completed both the training session and post-test. Their PGY training level was: PGY1 (38 %), PGY2 (25 %), PGY3 (25 %) or PGY4 (12 %). Procedural skill improved in 15/16 residents (paired *t*-test; *p* < 0.001), driven by a significant improvement in skill for residents in PGY1 (*P* = 0.015) and PGY2 (*p* = 0.003) but not PGY3 or PGY4. Overall anxiety scores were higher at baseline than at post testing (mean ± SD; 44.8 ± 12.1 vs 39.7 ± 9.4; NS) however only PGY1 residents experienced a significant reduction in anxiety (paired *t*-test, *p* = 0.04).

**Conclusion:**

LP simulation training combined with an interactive training session may be a useful tool for improving procedural competence and decreasing anxiety levels, particularly among those at an earlier stage of residency training.

## Background

Lumbar puncture (LP) is an important diagnostic and therapeutic tool. It is performed for the purpose of obtaining a sample of cerebrospinal fluid (CSF) [1]. In children, the most common reason for a LP is to diagnose an infection in the central nervous system (i.e. meningitis or meningoencephalitis). Lumbar puncture is also required for the diagnosis of many other non-infectious diseases in children such as inflammatory disease (e.g. transverse myelitis), hematological-oncological diseases (e.g. CNS leukemia or lymphoma) as well as subarachnoid hemorrhage or metabolic disease. In addition, LP is required to confirm CSF opening pressure to diagnose idiopathic intracranial hypertension. Less commonly, LP is required for therapeutic reasons to instill chemotherapeutic or antimicrobial agents directly into the CSF.

The Royal College of Physicians and Surgeons of Canada (RCPSC) and the Accreditation Council for Graduate Medical Education (ACGME) expect pediatric residents to become proficient at performing LP and to demonstrate effective, appropriate, safe and timely performance of this skill during their residency training [2, 3].

Residents have traditionally acquired LP skills by observing the procedure performed several times by a more senior colleague and then attempting the procedure themselves sometimes referred to as the “see one, do one, teach one” model of procedural skill teaching [3, 4]. This method of learning has been linked to variable procedural skill acquisition and resident self-reported lack of confidence and anxiety related to the supervision that they received [5].

Simulation-based training for procedural skills has been shown to be effective for trainees and has been widely used in many residency programs [6–9]. The aim of this study was to assess changes in pediatric resident procedural skill and self-reported anxiety using a LP simulator and an interactive training session. We hypothesized that use of a simulation-based model and an interactive training program would: 1) improve resident procedural skill as measured by a previously published 21-point scoring system [6] and; 2) decrease residents’ self-reported anxiety scores as measured by the standardized State-Trait Anxiety Inventory - State Anxiety Scale (STAI-S) questionnaire [10].

## Methods

All pediatric residents (*N* = 54) in post-graduate year (PGY) 1 to 4 who were based at the Children’s Hospital of Eastern Ontario in Ottawa, Canada were invited to participate. Residents could be part of the University of Ottawa program (*N* = 41) or rotating pediatric residents from the Northern Ontario School of Medicine Program (*N* = 13). Residents were excluded from the study if they had started any form of fellowship training.

Residents were each assigned a unique, confidential study number and completed testing one at a time. Residents were first asked to complete a brief questionnaire stating their: 1) PGY level; 2) gender and; 3) number of prior successful LPs that they have performed. Successful LPs were defined as a procedural attempt that obtained clear cerebral spinal fluid (CSF) and/or blood-tinged CSF that cleared. An unsuccessful LP was defined as failure to obtain CSF and/or bloody CSF that did not clear. Residents were then asked to complete the STAI-S immediately prior to entering the room to perform the LP. They were asked to consider the following when completing each question: “how you feel when performing a lumbar puncture”. The completed STAI-S was placed in a sealed envelope containing only their confidential study number attached. Data were later tabulated by the study coordinator who did not know the residents and had no role in their evaluation.

Immediately after completing the STAI-S, residents entered a room used for objective structured clinical examinations (OSCEs) and were asked to: “carry out all steps you would normally perform when completing a lumbar puncture on a 10 month old infant, including a check of cerebral spinal fluid opening pressure”. In this study, we used the Pediatric Lumbar Puncture Simulator (# KKM43C; Limbs and Things, Savannah, GA) that corresponded in size and appearance to a 7–10 month old infant. Residents were told that a nurse was available to assist during the procedure. They were informed that a sterile LP kit and other equipment were available for use (i.e. sterile gloves, lidocaine, needles, cleaning solution and a garbage can). The performance of each resident was scored by direct observation by an attending pediatric neurologist or attending pediatric intensive care physician using a previously published 21-item lumbar puncture skills checklist [6]. At the end of the LP, each resident was asked to list the tests that they would order on CSF analysis for an infant that was clinically suspected to have bacterial meningitis.

Within 1 month of completing the pre-test, the pediatric residents participated in an interactive teaching session about LPs as part of an academic half-day session. The session included a series of short videos demonstrating an LP performed by an attending physician. Residents were also provided with an opportunity to practice LP proficiency in small groups, using the pediatric lumbar puncture simulator, and to assemble the components of the LP test kit (i.e. manometer to check opening pressure, opening tubes, etc.) under the supervision of an experienced neurologist. All residents who completed the pre-test attended the teaching session.

Residents were then scheduled to complete a post-test (i.e. repeat STAI-S and observed LP) within 4 months of the teaching session.

A total of five attending physicians scored the procedural checklists. Three completed the pre-test scoring and three completed the post-test scoring. No resident had the same physician complete both his/her pre-test and post-test. All attending physicians scoring the resident LPs had more than 5 years of experience conducting and scoring resident OSCEs and all were Medical Educators and/or Program Directors at the University of Ottawa, Department of Pediatrics.

Data from the pre-tests and post-test were compared using paired t-tests with statistical significance set at *p* < 0.05. The IBM SPSS® Statistics 23 was used for all statistical analysis.

## Results

Twenty pediatric residents (20/54; 37 %) completed the pre-test (Sept 2014) and the interactive training session (Oct 2014). Sixteen residents (16/20; 80 %) completed the post-test (Dec 2014 to Feb 2015). Only the 16 pediatric residents who completed all three sessions, namely, the pre-test or baseline LP, teaching session and post-test or follow-up LP, were included in the analysis.

Most study participants were female (75 %). Post-graduate year of training corresponded to: PGY1 (*N* = 6; 38 %); PGY2 (*N* = 4; 25 %); PGY3 (*N* = 4; 25 %) and PGY4 (*N* = 2; 12 %). Prior to baseline testing, residents indicated the number of prior successful LPs (Table 1). No PGY1 residents reported any prior, successful LPs. The number of prior, successful LPs increased with advancing PGY of training.

**Table 1:** Resident baseline self-report of their prior successful lumbar punctures

Training year	Participant#	Number of prior successful lumbar punctures			
		0	<5	5–10	>10
PGY1	6	6			
PGY2	4		2	1	1
PGY3	4		1	1	2
PGY4	2				2

*PGY* post-graduate year of training

**Fig. 1:**
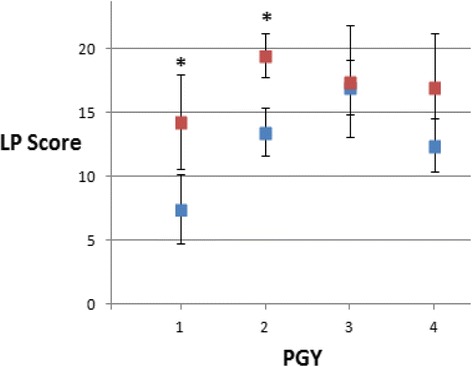
Resident lumbar puncture proficiency. Pediatric Resident lumbar puncture (LP) proficiency score on a 21-item LP skills checklist. Mean values (± standard deviation) are provided for each postgraduate year (PGY) of training. *Blue squares* indicate baseline score prior to the training session. *Red squares* indicate score at repeat testing after the training session. The asterix (*) denotes significant difference (paired *t*-test) seen in PGY1 (*p* = 0.015) and PGY2 (*p* = 0.003) but not in more senior years (PGY3; NS and PGY4; NS)

Overall, procedural skill improved in 15/16 residents from a mean pre-test score of 58 % to a mean post-test score of 80 % (paired *t*-test; *p* < 0.001). This improvement in procedural competency, as measured by an improvement in pre-test versus post-test LP checklist score, was driven by the improvement observed in more junior pediatric residents. Specifically, pediatric residents in PGY1 improved from a mean pre-test score of 35.7 % to a mean post-test score of 68 % (*p* = 0.015) while residents in PGY2 improved from a mean pre-test score of 67.8 % to a mean post-test score of 92 % (*p* = 0.003) (Fig. 1). No significant improvement was noted in PGY3 or PGY4 resident mean pre-test versus post-test LP checklist scores. We observed specific items on the LP checklist score to show particular improvement after the formal teaching session and opportunity for residents to practice their LP technique, specifically: use of lidocaine, manometer set up and opening pressure determination (Table 2).

**Table 2:** Procedural steps correctly performed in pre-test and post-test observation

Procedural steps^a^	Pre-test	Post-test
1. Consent obtained	37.5 %	68.8 %
2. Washed hands	87.5 %	87.5 %
3. Calls “time out”	0 %	31.3 %
4. Positions patient	81.3 %	100 %
5. Anatomical location	81.3 %	87.5 %
6. Sterile gloves on	93.8 %	93.8 %
7. Equipment set up	37.5 %	75 %
8. Clean skin	93.8 %	93.8 %
9. Drape patient	75 %	93.8 %
10. Lidocaine drawn up	6.3 %	87.5 %
11. Lidocaine injected	6.3 %	87.5 %
12. Needle orientation	75 %	87.5 %
13. Needle bevel correct	18.8 %	75 %
14. Needle slowly advanced	68.8 %	87.5 %
15. Opening pressure	37.5 %	62.5 %
16. Obtains and collects fluid	68.8 %	75 %
17. Re-inserts stylet at end	62.5 %	75 %
18. Dressing applied	86.7 %	100 %
19. Post-op orders written	18.8 %	31.3 %
20. Correct labs ordered	75 %	81.3 %
21. Sterile technique maintained	87.5 %	68.8 %
Overall average:	58 %	80 %

Procedural steps taken (i.e. % of Pediatric Residents (*N* = 16) correctly performing maneuver) on 21-item score on pre-test and post-test observation. ^a^This checklist of procedural steps has been previously by Barsuk et al. [6]

**Fig. 2:**
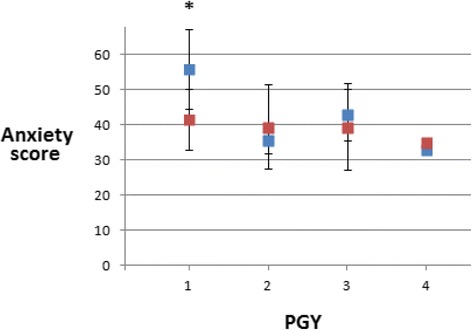
Resident self-report anxiety scores. Pediatric Resident self-reported anxiety scores using the “state” component of the State-Trait Anxiety Index (STAI). Mean values (± standard deviation) are provided for each postgraduate year (PGY) of training. *Blue squares* indicate the baseline scores prior to the interactive training session. *Red squares* indicate the scores at repeat testing after the training session. The asterix (*) denotes significant difference (paired *t*-test) within each PGY: PGY1 (*p* = 0.04); PGY2 NS; PGY3 NS, PGY4 NS

Resident self-report of anxiety decreased in a subset of more junior residents. Although the overall mean STAI-S anxiety scores were higher at baseline LP (mean = 44.8 ± 12.1) compared to the follow-up LP (39.7 ± 9.4), we did not observe a significant overall change in anxiety. However, PGY1 residents did show a significant reduction in self-reported anxiety between pre-test and post-test lumbar punctures (paired *t*-test, *p* = 0.04). This was not observed for pediatric residents in PGY2 or higher (Fig. 2).

In order to confirm that the PGY1 and PGY2 sample size was adequate, a post-hoc analysis was performed, calculating the mean of paired differences between pre- and post-simulation scores that would have been able to detect with our data set for the subset of participants in PGY1 and 2. Using the PASS 14 software [11], it was determined that our sample size of 10 PGY1 and 2 residents achieved 90 % power to detect a mean of paired differences of 4.1 with our observed standard deviation of differences of 3.6 and with a significance level (alpha) of 0.05 using a two-sided paired *t*-test. Given that the observed mean of paired differences for the PGY1 and 2 subsets was 6.3, with this power analysis, it was confirmed that the study was adequately powered to detect the difference that we observed in the subset of PGY1 and 2 residents.

## Discussion

This study demonstrates that an interactive teaching session and practice using a lumbar puncture simulator significantly improves procedural competence among junior (PGY1 and PGY2) pediatric residents and decreases self-reported anxiety among the most junior (PGY1) trainees. The improvement in resident procedural skill acquisition showed several findings. Each are of interest in the context of medical educational literature as it pertains to competency development for medical procedures.

The PGY1 residents completed their pre-test after only 2 months of training. None of the PGY1 residents reported any prior successful lumbar punctures. Given their stage of training it may not be a surprise that the most junior (PGY1) residents demonstrated the greatest improvement in procedural skill acquisition showing a mean improvement of 32.3 % in their pre-test versus post-test LP checklist scores. This finding was similar to that of Barsuk et al. [6] who first developed the 21-item lumbar puncture checklist. They followed 58 internal medicine residents (PGY1) using a similar pre-test versus post-test design. Their scores of the internal medicine residents showed a similar increase from 46.3 to 95.7 % after receiving a teaching session and feedback [6]. However, our study also identified a 24.2 % improvement in the pre-test versus post-test LP checklist scores for PGY2 pediatric residents which increased to a mean 92 % in post-test scores. Unlike the PGY1 residents, the PGY2 residents had reported some previously successful procedures (Table 1). This finding indicates that there may be educational value to using simulation training with residents in other post-graduate years, particularly those that may have had non-standardized training which may result in variability and/or perpetuation of mistakes and bad habits. The most senior (PGY3 and PGY4) residents did not demonstrate a significant change in their LP checklist scores which may be attributable to the larger number of procedures done by residents in these years.

The reduction in self-reported anxiety scores was significant only for PGY1 trainees. Given that this group of junior residents reported no prior successful LPs, we interpret this as a measure of initial resident discomfort and low self-confidence as they are asked to perform a complicated, multi-step procedure with which they have little or no familiarity. A survey of practicing physicians in the United Kingdom found that 42 % recalled feeling inadequately trained to perform procedures when they first began doing so independently [12]. These physicians cited a lack of formal and consistent training that has been a criticism of the former “see one, do one, teach one” method of acquiring procedural skills in medicine [12]. We postulate that the reduction in post-test anxiety among PGY1 residents is attributable to their increased exposure, standardized teaching and overall improved familiarity with lumbar punctures. We would favor the use of simulation for the initial attempts at such procedures to improve resident confidence while at the same time improving patient safety and reducing potential for medical error.

Manthey and Fitch [13] have proposed four stages for developing competency in medical procedures. Knowledge acquisition is the first stage and is characterized by the learner creating an appropriate framework of knowledge that will complement his or her future skill acquisition. Exposure is the second stage and is acquired by indirect observation and direct participation in the procedure. Skill acquisition is the third stage and occurs when the learner performs and practices a procedure. Assessment is the fourth and final stage that occurs when the trainee demonstrates competency in their medical knowledge and independent procedural skills [13]. We feel that our findings fit well into Manthey’s stages of developmental competency. The PGY1 residents, presumably at the earliest stage of knowledge acquisition, demonstrated the largest improvement in their pre-test versus post-test procedural ability scores as well as a reduction in self-reported anxiety. Interestingly, even though the PGY1 residents had the same interactive teaching session and equal opportunity to practice with the LP simulator, their final scores did not reach that of their PGY2 to PGY4 counterparts (Fig. 1). The PGY2 residents, having had some prior experience with LPs were likely at Manthey’s second or third stage of procedural skill acquisition. Their previous experience may have provided them with a conceptual framework to facilitate a higher overall skill acquisition and procedural competency score, as reflected in their mean post-test score of 92 % compared to their PGY1 colleagues that showed a mean post-test score of 67.8 %. We hypothesize that it is precisely the prior LP experience and familiarity that PGY2 to PGY4 residents had that accounted for their lower and stable pre and post-test anxiety scores.

Our study provides data that will be helpful for medical educators to decide how best to apply simulation-based procedural skills training across the PGY spectrum. Canadian residency training programs are in the process of moving towards a competency-based medical education (CBME) model [14]. As such, these results may be helpful with the planning of procedural skills training under this new education model. We strongly support the use of such simulation tools for first-year or new entry pediatric residents. Our study clearly shows that lumbar puncture simulation exercises have the greatest potential benefit for improving procedural competency among PGY1 pediatric residents. There is clearly an advantage for both residents and patients alike when junior pediatric residents are required to demonstrate basic procedural competency using a simulation device before moving on to attempt LP on an infant or a child. The previously reported 21-item LP checklist is a potential tool to evaluate trainees although there is a need to ensure that this validity evidence is established for this checklist and that all items are agreed upon by multiple residency programs, post-graduate medical education committees and/or examination boards.

Our results also indicate that there may be a role for simulation training in improving procedural competency among PGY2 pediatric residents. Despite the fact that PGY2 residents reported some prior successful LPs, they demonstrated a significant improvement in procedural competency following simulation training. It is possible that the PGY2 residents may have had variable teaching that can occur with procedural skill acquisition. Moreover, PGY2 residents are adding considerably to their depth of knowledge and given the clinical experience that they have gained over their first year of residency education, they may be at an ideal stage to enhance and retain such procedural skill. As such we recommend simulation based LP teaching sessions annually for at least PGY1 and PGY2 residents. More senior residents may be excused from such simulation training if they have been formally assessed by staff physicians and found to demonstrate competency in their medical knowledge and procedural skills in both simulation and patient-procedures.

Our study re-tested residents over a short time-frame and did not assess to what degree their procedural skills were retained. It also did not examine if repeat or refresher training would be advantageous. Other studies have demonstrated that simulation training skills using central line placement by internal medicine residents are indeed retained when residents are re-tested 6 and 12 months post training [15].

In addition to the move towards CBME, there are additional reasons why the use of simulation-based learning for lumbar punctures may be of increasing importance. As the incidence of childhood meningitis has decreased in recent decades [16] as a result of conjugate vaccines there may be less opportunity for pediatric residents to perform lumbar punctures. The very appropriate increase in focus on patient care and safety has also made the traditional method of learning less acceptable. Like all procedure skills, experience is paramount for success. One adult study examined unsuccessful lumbar punctures by neurologists and found that inexperience was a greater cause of failed LP attempts as compared to all other causes [17].

Learning procedures through a controlled, simulated experience ensures that residents are taught the proper techniques including equipment set-up, patient positioning and assessment of risk [6, 18, 19]. Improved procedural skills have also been shown to persist among residents with continued skill improvement upon re-testing as long as 6 months after the initial simulation based training [18]. Junior residents have a great deal to gain from simulation-based experiences and simulation-based mastery learning (or intern “boot-camp”) can have a positive impact on resident performance [20]. Permitting residents to complete a “practice run” using a simulator provides them with an opportunity to review these aspects of the procedure in a relaxed and safe environment and better anticipate what to expect when the procedure is replicated on a patient. We recommend the use of such an itemized checklist when residents are transitioning from simulation-based practice to patient-based care to ensure that the same rigorous process is followed and patient safety is optimized.

Our study does have limitations. First, the small sample size may limit our ability to generalize our findings. It is unknown if those who participated are more or less likely to be proficient at LPs than the residents who did not participate. Second, variability can exist even among experienced OSCE examiners. Simultaneous assessment by two attending physicians and/or blinded review of video recorded sessions are two examples of ways that future projects could aim to evaluate inter-examiner variability. In addition, this study examined the value of LP simulation models and an interactive teaching session, so the improvement observed in the procedural skill and comfort of junior residents cannot be attributed to one item in isolation. Finally, we did not assess skill retention over time or LP performance on real patients in a clinical setting.

We would encourage future research projects aimed at examining whether or not repeat or refresher simulation training would be advantageous for pediatric residents. We would also encourage the collection of validity evidence for the previously reported 21-item LP checklist for use in infants and children by appropriate pediatric residency programs, post-graduate medical education committees and/or examination boards. We infer that more procedural training and experience, whether on simulated or real patients, should improve clinical outcomes. We believe that the use of simulation not only offers advantages for patient safety but also potential advantages for residents if anxiety is reduced and/or self-confidence is improved. Such answers will be important not only for integrating this into emerging CMBE curriculums but also ensuring any questions regarding the need for long-term procedural skill competency (through continuing medical education programs) are properly considered.

## Conclusion

This study provides evidence that the combined use of a LP simulator and interactive teaching session has a positive impact on simulated procedural competence and self-reported anxiety in junior pediatric residents.

## Abbreviations

ACGME, accreditation council for graduate medical education; CBME, competency-based medical education; CSF, cerebral spinal fluid; LP, lumbar puncture; OSCE, objective structured clinical examinations; PGY, post-graduate year; RCPSC, Royal College of Physicians and Surgeons of Canada; SD, standard deviation; STAI-S, State-Trait Anxiety Inventory – State Anxiety Scale
